# Piloting real-time assessment of movement behaviors in adults with mobility disabilities: a feasibility study

**DOI:** 10.1016/j.dhjo.2026.102173

**Published:** 2026-09-29

**Authors:** Chelsea L. Kracht, Annie M. Rice, Taylor Collier, Richard A. Washburn, Joseph E. Donnelly, Anna M. Gorczyca

**Affiliations:** Division of Physical Activity and Weight Management, Department of Internal Medicine, The University of Kansas Medical Center, 3901 Rainbow Boulevard, Kansas City, KS, 66160, USA

**Keywords:** Momentary assessment

## Abstract

**Background:**

Adults with mobility disabilities (MD) experience significant health disparities, including high rates of obesity and low levels of physical activity, due to a range of persistent barriers. Addressing these challenges requires innovative strategies that capture real-world behaviors and contexts to inform tailored interventions.

**Objective:**

This pilot study evaluated the feasibility and acceptability of ecological momentary assessment (EMA) to assess movement behaviors in adults with overweight or obesity and a MD.

**Methods:**

Twenty-four adults with overweight or obesity (body mass index >25 kg/m^2^) and a permanent MD participated in a 5-week observational study. The EMA protocol included two 7-day assessment periods separated by a 3-week no-contact interval. Participants completed four EMA surveys/day (morning, evening, and two signal-contingent) via smartphone. Demographics and anthropometrics were self-reported at baseline. Movement behaviors were assessed via wrist-worn accelerometer. Feasibility of the EMA protocol was defined as completion of >75% of prompts and a mean feasibility questionnaire score of ≥4.0 on a 5.0-point scale. Acceptability was measured via questionnaire on a 5.0-point scale.

**Results:**

Participants (~55.2 ± 13.3 years, 79% female) completed 303 morning, 586 signal-contingent, and 303 evening surveys. Mean moderate-to-vigorous physical activity was 36.6 ± 27.3 min/day; total physical activity was 147.4 ± 76.3 min/day. EMA compliance was 91.6%, exceeding feasibility criteria. Questionnaire results indicated high feasibility (4.6 ± 0.4) and acceptability (4.5 ± 0.5).

**Conclusions:**

EMA of movement behaviors is feasible and acceptable in adults with MD and overweight or obesity. These findings support the use of EMA in future behavioral interventions targeting movement and health outcomes in this underserved population.

## 1. Introduction

Adults with mobility disabilities (MD) represent a large segment of the U.S. population with disproportionately higher rates of obesity (~42%) and lower levels of physical activity (PA) compared to non-disabled adults, resulting in increased chronic disease risk.^1,2^ Further compounding these disparities, individuals with MD face persistent environmental, physiological, and social barriers that undermine engagement in physical activity and dietary self-management.^3,4^ Innovative strategies tailored to the unique challenges of adults with MD are urgently needed to reduce health risks and promote sustainable behavior change.

Meeting the World Health Organization’s recommendation of 150–300 min per week of moderate-to-vigorous physical activity (MVPA) is often unrealistic for adults with MD, given fluctuations in pain, fatigue, and functional decline.^5,6^ A more feasible and potentially effective^7^ strategy is to target the full spectrum of daily movement behaviors, i.e., sleep, sedentary time, light physical activity, and MVPA^8^ rather than focusing on a single behavior, e.g., increasing MVPA.^2^ Improvements across these behaviors may contribute to weight management and reduced chronic disease risk, even independent of weight loss.^9^

Ecological momentary assessment (EMA) is a real-time data capture method that may be well-suited to capture movement behaviors and the context in which they occur for adults with MD. Using smartphone technology, participants are prompted at selected time points during the day to respond to a brief set of questions regarding experiences and contextual information.^10^ EMA has been used to collect real-time data including emotional state, physical symptoms, and activities of daily living in both healthy and clinical populations including individuals with MD, but not yet movement health behaviors.^11–13^ EMA has been widely used to study movement behaviors in other populations, including within and across day changes and social cognitive determinants of movement across the lifespan.^14–16^ EMA efficiently captures movement behaviors and their contexts including location, social setting, barriers and facilitators (e.g., mood, pain, weather), and sleep, information that is burdensome or unavailable with self-monitoring methods such as paper diaries or activity trackers. Data collected from EMA can be used to tailor subsequent behavioral interventions^17^ or to inform the development of ecological momentary interventions.^18^

EMA may be especially useful for identifying changes in health, physical function, pain etc. frequently experienced by adults with MD which may impact their ability to engage in specific types of physical activity. Utilizing EMA-informed behavioral counseling would allow interventionists to provide feedback that is comprehensive, autonomy-supportive, and responsive to real-life barriers and facilitators. However, we are unaware of trials that have evaluated the effectiveness of EMA informed behavioral counseling to improve movement behaviors in ambulatory adults with MD and overweight or obesity who do no use any type of wheeled mobility for primary locomotion in the context of a weight management intervention. Therefore, we conducted a pilot study to evaluate the feasibility, acceptability, and appropriateness of our smartphone-delivered EMA protocol focused on movement behaviors in a volunteer sample of 24 adults with a permanent MD and overweight and obesity. We also assessed the feasibility of using wrist worn accelerometry to assess movement behaviors in this sample.

## 2. Methods

### 2.1. Overview

This pilot study assessed the feasibility of an EMA protocol (1-week of EMA, 3-weeks of no EMA, and a final week of EMA) in 24 ambulatory adults with a MD who were willing to download a mobile app on their phone. This one-arm pilot study was approved by the [University] institutional review board (STUDY00161579) and conducted between May 2025 and July 2025. The current analysis follows EMA reporting guidelines, the Checklist for Reporting Ecological Momentary Assessment Studies (CREMAS, Supplemental Table 1).^19^

### 2.2. Participants

Participants were recruited using existing MD list serves and contact with adults with MD that had completed a previous trial conducted (DK116669). Inclusion criteria were: adults (≥18 years of age) with a permanent impairment (≥5 years) that resulted in a MD as defined by self-reported complications with one or more of the following criteria with or without assistive devices: walking (some or much), walking 0.25 miles without stopping, remaining on feet for >2 h, taking 10 uninterrupted steps, kneeling, stooping or crouching, or standing up from an armless chair. Participants were excluded if they self-reported a body mass index (BMI, kg/m^2^) below 25 kg/m^2^, using a wheelchair (manual or power) or power scooter as their primary assistive device, unwilling to download the mobile app or travel to research facilities for one in-person study visit. Participants were provided up to $100 for their research participation across the study.

### 2.3. Procedures

Research staff contacted eligible participants and invited them to an ~90 min visit at our on-campus research facility. At visit 1, research staff explained the study protocol, answered questions and obtained written informed consent prior to data collection. Participants then completed baseline surveys using REDCap electronic data capture tools hosted at [University].^20,21^ Following survey completion, accelerometry procedures utilizing a wrist-worn device (Model wGT3x-BT triaxial accelerometer, ActiGraph LLC, Pensacola, FL) were explained, and an EMA orientation was conducted. All participants used their own smartphones for the EMA surveys. Research staff downloaded the LifePak app (Life-Data Corporation, Marion, IN) onto the participant’s phone which is compatible with both Apple^®^ and Android iOS^®^ devices. The LifePak app delivers phone notifications as reminders and allows EMA survey completion within the app. Staff reviewed the prompting schedule, any phone capabilities that may limit receiving messages (e.g., “do not disturb” times), and the EMA questions and response options with participants. Participants were provided an opportunity to ask questions about the software. After downloading the EMA app, the participant began receiving surveys.

Visits 2-4 were conducted via Zoom. Visit 2 was conducted approximately 7 days after the completion of visit 1. Participants were instructed how to remove the EMA app from their phone and a reminder to return the accelerometer using the provided prepaid envelope. After completion of visit 2, participants had a 3-week no-contact period. The purpose of this no-contact period was to simulate a monthly EMA schedule which could be used in future trials to reduce participant burden. During visit 3, participants were instructed to redownload the EMA package to the mobile app on their personal device, which would initiate the second round of EMA surveys for 7-days. Participants also scheduled their final visit (visit 4) approximately one week after the completion of visit 3. Prior to visit 4, participants were sent a weblink via email to complete the feasibility and acceptability survey. During visit 4, participants were instructed to delete the mobile app from their personal device completely. They were also given time to complete feasibility and acceptability surveys if necessary.

### 2.4. Ecological momentary assessment

Participants were asked to complete up to four EMA surveys/day, including two signal-contingent surveys and two fixed surveys (a morning and evening survey). Signal-contingent or “surveys occurred randomly within fixed 4-h windows (session 1: 8:00am–12:00pm and session 2: 4:00-8:00pm) and expired 2-h after the initial reminder. The morning survey was available for 4 h (8:00-12:00pm), as was the evening survey (6:00-10:00pm). Participants were given up to three reminders for signal-contingent surveys, including the initial reminder, and reminders at 30-min, 60-min, and 90-min after the initial reminders, if necessary. Participants were sent reminders for the morning and evening surveys every hour, if necessary. The EMA software measured completion time from opening the survey to completion of final question. These sampling time frames and reminders^22^ and similar prompting strategies (<5 times/day) are recommended from other EMA studies.^23^

### 2.5. Outcomes

#### 2.5.1. Demographics

Participants self-reported demographic characteristics which included age, race/ethnicity, marital status, employment status, education level, and household income as well as height and weight using electronic database.^20,21^

#### 2.5.2. EMA measures

The study team reviewed the existing literature and barriers to PA identified in our previous trial of adults with MD (NCT04046471, n = 129) when selecting EMA measures. EMA measures were reviewed by our community advisory board that included four adults with MD, who did not participate in the study. The community advisory board also provided feedback on the EMA response options and other descriptions used in the study. Questions occurred in the same order for all surveys. The list of items included in the surveys are provided as supplementary material (Supplementary Table 2–4).

In the morning survey, participants were asked five questions, based on the previous night, how many hours of actual sleep they received, bed-time and wake-time, and overall sleep quality. These questions were adapted from the Pittsburgh Sleep Quality Index, which is validated in adults.^24^

The signal-contingent surveys assessed participant location and level of activity. Participants who reported exercise were further queried regarding exercise type, whether they are exercising alone or with someone else (who), and exercise intensity. These surveys were based on other EMA studies of momentary physical activity.^25,26^

The evening survey addressed daily barriers and facilitators to physical activity. In separate questions, participants were asked if they engaged in light, moderate, and vigorous physical activity, and if so, for how many minutes during the day. For each activity intensity, participants were provided one sentence guidance to help calculate their activity during the day. These questions were based off the Godin-Shepard leisure questionnaire, a validated questionnaire of weekly physical activity.^27^ Barriers and facilitators of physical activity in MD responses were based on qualitative data collected in our recently completed trial (DK116669).^28^ Barrier options included “too tired”, “feeling what I do doesn’t help”, “impairment/pain”, “no one to help me”, “not interested”, “embarrassment about my appearance”, “lack of time”, “bad weather”, “other”, “N/A was active today”. Facilitator options were “free time”, “being well rested, “pain relief”, “people to exercise with”, “assistance”, “mood and exercise enjoyment”, “encouragement from others”, “weather”, “activities of daily living (i.e. housework, yardwork, etc.)”, “manual labor job”, “other”, and “N/A no facilitators”. For barriers, participants were asked which barriers they faced that day and asked to select all that apply. In a separate question with the same response options, participants were asked to identify the major barrier to activity that day. Similar question format was conducted for facilitators, including a question assessing all facilitators and the major facilitator that day. Additional details on barrier and facilitator questions can be found in Supplementary Table 3.

#### 2.5.3. Feasibility, acceptability and appropriateness

Feasibility was defined by the total compliance (number of EMA prompts answered divided by total prompts delivered), individual session compliance, and feedback on device usability and EMA schedule. Feasibility, acceptability, and appropriateness were assessed using an online survey administered at Visit 4 based on an existing validated questionnaire for intervention outcomes.^29^ This questionnaire included subscales of feasibility of intervention measure (3 questions), acceptability of an intervention measure (3 questions), and intervention appropriateness measure (2 questions). The feasibility questionnaire inquired about the ease of reporting the content of surveys, number of surveys, and timing of surveys. Acceptability included the approval of the content of surveys, number of surveys, and timing of surveys. Appropriateness inquired about using their personal phone to answer questions and applicability of survey content. Responses for each subscale were averaged for an overall score (range: 1-5, with higher being more feasible/acceptable/appropriate).^29^

#### 2.5.4. Movement behaviors and sleep

Movement and sleep behaviors were assessed using a wrist-worn accelerometer on the non-dominant wrist as determined by self-report of handedness. Participants were asked to wear the accelerometer over 7 consecutive days except when engaging in water-based activities, e.g., showering, swimming, etc. Accelerometers were initialized to collect and store raw acceleration data at 30 Hz using ActiLife software version 6.13.4 (AcitGraph LLC). Accelerometer data were collected at 30 Hz and processed using the GGIR package (3.2-6),^30,31^ in R to compute Euclidean Norm Minus One (ENMO) in milli-gravitational units (mg). The GGIR package first detected non-wear time defined as 15-min blocks where the standard deviation of two out of three axes was <13 mg or the range <50 mg. Remaining time was then classified into movement behaviors of sleep, sedentary time (inactive time), light PA, moderate PA, and vigorous PA. First, sleep was calculated based on angle variability, and total sleep time, or time spent in “sleep” regardless of duration was reported.^30^ PA metrics were then calculated using ENMO metrics of inactive (sedentary time, 0-39 mg), light PA (40 mg ≤ ENMO <100 mg), and MVPA (ENMO ≥100 mg).^32^ Bouts of ≥1 min where 80% of the activity was classified as MVPA were used to reduce the impact of random wrist movements.^33^ Days with >10 h of awake wear and ≥20 h of total wear time (awake and sleep) were considered a valid day. Bouts of sleep >180 min were considered, and participants with ≥1 valid day were reported. Variables were summarized for each participant (minutes/day).

### 2.6. Data analysis

All available data are reported. Descriptive statistics were used to describe the baseline characteristics of the study sample with means ± standard deviations for continuous variables and frequency (percentages) for categorical variables. EMA compliance is operationalized as the percentage of EMA survey prompts completed out of all prompts sent. Our a priori threshold for feasibility was defined as overall compliance was >75% of prompts. Feasibility, acceptability and appropriateness were also assessed as an average of the Likert scale questions (range: 1-5). An overall rating of ≥4.0/5.0 was deemed feasible, acceptable or appropriate, respectively. The data processing was conducted using SAS version 9.3 (Cary, N.C.).

## 3. Results

Fifty-three individuals completed the initial eligibility questionnaire, 32 were eligible and invited to participate, 24 individuals were consented, and 23 individuals completed the entire study. One individual was lost to follow up after the initial EMA period. As shown in Table 1, participants were 55.2 ± 13.3 years old, 79% identified as female, with 30% non-White and 8% Hispanic representation. Disability cause was primarily (62.5%) health impairment, rather than congenital (25%) or injury/amputation (12.5%). Self-reported data revealed a BMI of 38.3 ± 8.6 kg/m^2^, with a third (33.3%) experiencing class III obesity. Individuals were primarily married (45.8%), had a college degree (29.5%) or some college (29.1%), and had a middle household income (33.3%).

### 3.1. Feasibility and acceptability

In total 1282 surveys (638 fixed and 639 signal-contingent) were delivered, and 1175 of them were completed (overall compliance: 91.65%). Compliance rates were 93.5%, 88.1%, 89.9%, and 94.9% for the morning, session 1, session 2, and evening surveys, respectively (Table 2). Fixed surveys were completed within an average of 2 min and 11 s, while signal-contingent surveys were completed within 47 s. Overall, participants indicated that the EMA protocol was feasible (4.6 ± 0.4), acceptable (4.5 ± 0.5), and appropriate (4.5 ± 0.7).

### 3.2. Movement behaviors and sleep in adults with MD

Fifteen participants contributed an average of 6.7 ± 0.8 valid days wearing the accelerometer during week 1 (Table 3). The eight accelerometers not returned include three with initialization errors, four with errors in data retrieval, and one was broken. On average, participants accumulated 36.4 ± 27.0 min/day of moderate activity and 0.2 ± 0.4 min/day of vigorous activity, with most of their time was spent in inactive/sedentary time (85%, 14.3 h/day). Based on accelerometry, participants spent most of their sleep period in sleep (86 ± 8%) rather than awake, and average 6.6 ± 1.6 h of total sleep time.

### 3.3. Barriers and facilitators to movement behaviors

The major barriers to PA reported by participants were impairment/pain, other, and work/home responsibilities (Table 4). When reporting all barriers, one third of participants reported they were active that day, so they experienced no barriers (*n* = 99, 32.3%). The remainder who noted barriers reported mainly pain (*n* = 81, 26.4% of surveys), work home responsibilities (*n* = 81, 26.4% of surveys), other barrier (*n* = 70, 22.8%), not interested (*n* = 57, 18.5%), too tired (*n* = 46, 14.9%), with all other options less frequently reported (<5%). The major facilitators of PA were activities of daily living (22.3%) and free time (14.4%) however not having any facilitators (27.3%) was the most prevalent (Table 4). When considering all possible facilitators reported, individuals mainly reported activities of daily living (33.4%), free time (27.5%), people to exercise with (12.1%), weather (12.7%), pain relief (11.1%), being well rested (10.1%), encouragement from others (9.8%), mood (8.8%) and all other options were less commonly reported (<5%).

## 4. Discussion

This study evaluated the feasibility and acceptability of a real-time assessment modality to capture movement behavior in individuals with MD. Participants reported the modality was both feasibility and acceptable, which was confirmed by compliance rates above feasibility thresholds. These EMA surveys also captured daily patterns of health behavior, demonstrating individuals with MD were predominately sedentary when answering the surveys, achieved PA through activities of daily living, but still faced multiple barriers but to being active during the day. This study contributes to the evidence that EMA is a capable modality to capture PA in adults with MD.

At two separate time points, EMA completion rates demonstrated this modality was highly feasible in adults with MD. Our completion rates (~89-91%) exceed other compliance measures of systematic reviews in adults EMA studies (81.9%, 168 studies).^11^ The current study’s high completion rate may be attributed to the low number of surveys (<5 surveys) and few items per surveys (<20) which may positively impact compliance in non-clinical studies.^11^ This study did not provide compensation for completing surveys, as this incentive does not always translate into higher completion rates. The older age (~55 years) of our sample may have positively contributed to compliance rates as other EMA studies demonstrate older individuals have higher compliance rates relative to younger individuals.^34^ However, our sample size limits our ability to test such factors in the current study, but should be explored in future EMA studies in adults with MD.

The prompting schedule, content, and overall study conduct was deemed feasible and acceptable. These results may be attributed to the involvement of our community advisory board of individuals with a MD for study conduct, reviewing EMA survey questions and response options, as well as recruitment queries. Our results are in alignment with Silveira et al., who underscored the invaluable contribution of community advisory board in refining intervention content and ensuring appropriateness for adults with MD.^35^

The feasibility of EMA, coupled with obtaining real-time barriers and facilitators to PA is promising for future interventions. Pain was the predominant barrier identified by our sample, and aligns with previous single assessment research.^6^ Our results also found home/work responsibilities as a significant barrier to PA while the primary facilitator was activities of daily living, providing further evidence that promoting improvements in movement behaviors may be more feasible in this population.^7,8^ However, the interpretation of activities of daily living as a facilitator warrants further exploration. It is unclear whether participants viewed these activities as generating meaningful physical activity themselves or whether successfully completing daily tasks created opportunities, confidence, or motivation to engage in additional movement behaviors. Intervening in real-time or with behavioral counseling may be a potential avenue to reduce these barriers and facilitate beneficial amounts of movement behaviors.

Strengths of the current pilot study include testing a novel real-time capture of movement behaviors in a priority population, including a variety of MDs, incorporating a community advisory board to improve the internal validity. Limitations of this study include generalizability and pilot study nature. Compliance in this population was notably high but may not apply to all adults with MD. Nevertheless, the current study utilized a standardized protocol to increase EMA compliance. While this pilot study assessed EMA compliance, feasibility and acceptability, results may vary in a larger and longer trial.

## 5. Conclusions

The current pilot study results pose multiple future directions for research and practice. First, these compliance and PA barrier and facilitator results require replication in a larger, adequately powered trial. It is unclear if compliance rates may change across a longer trial with monthly prompting. Moreover, additional data on PA barriers and facilitators across the seasons and the year may provide a more comprehensive picture of adults of MD’s physical activity. Second, this future trial may consider using information captured from EMA to prompt PA, fruit and vegetable intake, and healthy sleep practices in a just-in-time adaptive intervention. The intervention may provide tailored real-time support based on participant-provided information,^17^ and possibly integration of wearable sensors (e.g., Fitbit) for momentary prompting.

In conclusion, this pilot trial found that EMA was feasible and acceptable to assess movement behaviors in adults with MD. The current results suggest EMA as an assessment, and potentially intervention modality may be well accepted amongst individuals with MD, with the potential to improve movement behaviors for future benefit.

## Supplementary Material

Supplemental

## Figures and Tables

**Table 1:** Demographic characteristics (n = 24).

	Mean ± SD	n	%
Age	55.2 ± 13.3		
*18- <65 years*		17	70.8
≥ *65 years*		7	29.2
Female		19	79.2
Ethnicity			
*Hispanic or Latino*		2	8.3
*Not Hispanic or Latino*		20	83.3
*Other or Unknown*		1	4.2
*Prefer not to answer*		1	4.2
Race			
*Black or African American*		5	20.8
*White*		17	70.8
*More than one race*		1	4.2
*Prefer not to answer*		1	4.2
Body Mass Index	38.3 ± 8.6		
*Overweight*		5	20.8
*Obesity, Class I*		5	20.8
*Obesity, Class II*		6	25.0
*Obesity, Class III*		8	33.3
Disability Type			
*Health Impairment*		15	62.5
*Congenital*		6	25.0
*Injury/Amputation*		3	12.5
Marital Status			
*Single, never married*		4	16.7
*Single, divorced*		6	25
*Single, widowed*		2	8.3
*In a domestic partnership*		1	4.2
*Married*		11	45.8
Education			
*Completed high school*		2	8.3
*1-3 years of college*		5	20.8
*Associate degree*		2	8.3
*Bachelor degree*		9	37.5
*Graduate degree*		6	25.0
Employment			
*Not employed*		14	58.3
*Employed, working 1-20 h*		1	4.2
*Employed, working 20-39 h*		1	4.2
*Employed, working 40*+ *hours*		8	33.3
Household income			
*Less than $20,000*		2	8.3
*$20,000-40,000*		3	12.5
*$40,001-60,000*		8	33.3
*$60,001-80,000*		2	8.3
*$80,001-100,000*		1	4.2
*$100,001-150,000*		6	25.0
*Greater than $150,000*		1	4.2
*Prefer not to say*		1	4.2

**Table 2:** Compliance with EMA surveys by delivery and week (*n* = 1175 surveys).

	Delivery	Time available	Week 1	Week 4	Overall
**Morning Survey**	Fixed	8:00am – 12:00pm	94.4%	92.6%	93.5%
**Session 1 Survey**	Signal-contingent	8:00am - 12:00pm	87.5%	88.8%	88.1%
**Session 2 Survey**	Signal-contingent	4:00pm - 8:00pm	90.5%	89.3%	89.9%
**Evening Survey**	Fixed	6:00pm – 10:00pm	94.3%	95.6%	94.9%

EMA = ecological momentary assessment; compliance indicates completion of entire survey.

**Table 3:** Movement behaviors of individuals with mobility disabilities (n = 15).

	units	Mean ± SD
**Wear-time**	*hours/day*	17.0 ± 2.7
**Number of Valid days**	*days*	6.3 ± 1.4
**Sedentary Time**	*min/day*	873.3 ± 178.5
**Light Physical Activity**	*min/day*	110.7 ± 52.2
**Moderate Physical Activity**	*min/day*	36.4 ± 27.0
**Vigorous Physical Activity**	*min/day*	0.2 ± 0.4
**Moderate-Vigorous Physical Activity**	*min/day*	36.6 ± 27.3
**Total Physical Activity**	*min/day*	147.4 ± 76.3
**Total Sleep Time**	*hours/night*	6.6 ± 1.6
**Sleep Efficiency** ^a^	*%*	86 ± 8

a Proportion of sleep period spent in total sleep.

**Table 4:** Fixed Survey Responses (n = 606, 24 individuals).

	Week 1	Week 4	Overall
	n (%)	n (%)	n (%)
**Major Barriers to Physical Activity**			
*No barriers, was active*	40 (26.5)	59 (37.8)	99 (32.5)
*Impairment/pain*	23 (15.2)	33 (21.2)	56 (18.2)
*Other*	22 (14.5)	25 (16.0)	47 (15.3)
*Work/home responsibilities*	28 (18.5)	18 (11.5)	46 (15.0)
*Not interested*	15 (9.9)	14 (8.9)	29 (9.4)
*Too tired*	19 (12.5)	9 (4.5)	26 (8.5)
*Bad weather*	4 (2.6)	0	4 (1.3)
**Major Facilitators to Physical Activity** ^a^			
*No facilitators*	40 (26.7)	43 (27.9)	83 (27.3)
*Activities of daily living*	35 (23.3)	33 (21.4)	68 (22.3)
*Free time*	19 (12.6)	25 (16.2)	44 (14.4)
*Other*	10 (6.6)	20 (12.9)	30 (9.8)
*Weather*	11 (7.3)	5 (3.2)	16 (5.2)

a All other facilitators were less commonly reported as major facilitators (<5% of responses) including being well rested, pain relief, people to exercise with, assistance from others, mood, and encouragement from others.

## Appendix A. Supplementary data

Supplementary data to this article can be found online at https://doi.org/10.1016/j.dhjo.2026.102173.
