# Supplementary material for: Piloting real-time assessment of movement behaviors in adults with mobility disabilities: a feasibility study
Source: Disabil Health J. Author manuscript; Available in PMC 2026 Nov 5. (PMC13738186; doi:10.1016/j.dhjo.2026.102173)
Supplement: Supplementary file 1 [file NIHMS2217028-supplement-Supplemental.docx]

**Supplementary Table 1. Checklist for Reporting Ecological Momentary Assessment Studies**

|  |  | **Item Number** | **Recommendation** | **Line** |
| --- | --- | --- | --- | --- |
|  | Title | 1 | Include ecological momentary assessment in title and key words | Title, Keywords |
| **Introduction** | | | | |
|  | Rationale | 2 | Briefly introduce the concept of EMA and provide reasons for utilizing EMA for this study or topic of interests (eg, to examine time-varying predictors of unhealthy eating occasions in children’s daily lives) | Introduction, Line 42-68 |
| **Methods** | | | | |
|  | Training | 3 | Indicate if, and by what methods, training of participants for EMA protocol was used | Methods, Line 92-102 |
|  | Technology | 4 | Describe what technology, if any, was used. Include the following information: device (eg, mobile phone, portable computer), model (eg, Nexus 4, iPod), operating system (eg, Android, Windows), and EMA program name | Methods, Line 92-102 |
|  | Wave duration | 5 | State the number of waves for the study (eg, 2 monitoring periods over the course of 1 year) | Methods, Participants, Line 88-102 |
|  | Monitoring period | 6 | State the number of days each wave of the study lasted, and how many weekdays versus weekend days | Methods, EMA, Line 118-127 |
|  | Prompting design | 7 | Indicate the prompting strategy used for the study (eg, event-based, interval-based, or a combination of the two). If using interval-based strategy, indicate what type of schedule is used (eg, fixed, random, or hybrid interval) | Methods, EMA, Line 118-127 |
|  | Prompt frequency | 8 | Intended frequency of prompts per day. Break down by weekdays and weekend days if applicable | Methods, EMA, Line 118-127 |
|  | Design features | 9 | Describe any design feature to address potential sources of bias (eg, reactivity) or participant burden (eg, EMA questions appearing in different orders) | Methods, EMA Line 118-127  Supplementary Table 2-4 |
| **Results** | | | | |
|  | Attrition | 10 | Indicate participant attrition throughout the study; report attrition rates both by monitoring days and waves, if applicable | Results, Line 211-219 |
|  | Prompt delivery | 11 | Report number of EMA prompts that were planned to be delivered. If possible, also report the number of EMA prompts that were actually received by participants and indicate reasons for why prompts were not sent out (eg, technical issues or participant noncompliance reason such as phone was powered off) | Results, Line 220-226 |
|  | Latency | 12 | Report the amount of time from prompt signal to answering of prompt | Results, Line 223-225 |
|  | Compliance rate | 13 | Report total answered EMA prompts across all subjects and the average number of EMA prompts answered per person. Report compliance rate both by monitoring days and waves, if applicable. Indicate reasons for noncompliance, if known | Results, Line 220-226, Table 2 |
|  | Missing data | 14 | Report whether EMA compliance is related to demographic or time-varying variables | Results, Line 235-247 |
| **Discussion** | | | | |
|  | Limitations | 15 | Discuss limitations of the study, taking into account sources of potential bias when using EMA methods (eg, reactivity, use of technology) | Discussion, Line 285-289 |
|  | Conclusions | 16 | Provide a general interpretation of results and discuss the benefits of using EMA (eg, improving understanding of daily behaviors) | Discussion, Line 292-304 |

| **Supplementary Table 2. Morning Survey Questions** | | |
| --- | --- | --- |
| - Available: 1 time per day, 8:00am-12:00pm - Overview: Daily Diary Questions and Answers related to sleep from previous day - Completion: Participants can go back through survey after starting | | |
| **Question** | **Answers** | **Description for participant** |
| Good morning, we would like to learn more about YOUR sleep habits LAST NIGHT.  1. What time did you fall asleep last night? | DATE ____: ____ AM/PM  *(note in LifeData this includes a date with time, so the participant can select times beyond 12:00am)* | The participant will list the time that they fell asleep. Fell asleep means when they went to sleep, rather than got in bed to sleep, which may be an earlier time. If the participant is having trouble figuring out the exact time they went to sleep, try to think about which half hour it is closer to. For example, was it closer to 10:00pm or 10:30pm. If the participant has trouble entering the date, ensure the time is correct. |
| 2. Did you wake up during the NIGHT? (e.g., go to the bathroom, another disturbance) | - Yes -> Next question - No - Don’t Know | Was there any time that the participant woke up during the night? |
| (if participant responds yes to #2)  2a. how long were you awake LAST NIGHT (e.g., time spent going to the bathroom or address disturbance)? | ___Hours___minutes  *(Life data scroll option for each, 0-23 hours, 0-59 minutes)* | This question asks how long they were awake for, which may range from minutes to hours. We are only counting items where you woke up for more than 5 minutes. |
| 3. What time did you wake up this morning? | DATE ____: ____ AM/PM  *(note in LifeData this includes a date with time, so the participant can select times beyond 12:00am)* | Time the participant woke up in the morning. If the participant has trouble entering the date, ensure the time is correct. |
| 4. How much *actual* sleep do you think YOU got LAST NIGHT? *(This may be different than the number of hours you spent in bed)* | ___Hours___minutes  *(Life data scroll option for each, 0-23 hours, 0-59 minutes)* | This is the amount of time the participant was **actually** asleep, which would exclude time from the total if they woke up at night, and from when they got in the bed (which is not necessarily sleeping). |
| 5. How would you rate your sleep quality overall from LAST NIGHT? | - Very bad - Fairly bad - Fairly good - Very good | The participant should rate their quality of sleep from last night using the prompts. |
| 6. Did you wear the tracker while you were sleeping last night? | - Yes - No - Don’t Know | The participant should report if they wore the activity tracker for the full night. **If there is difficulty wearing the tracker, please contact us.** |
| **Thank you for completing the survey!** | None |  |

| **Supplementary Table 3. Evening Survey Questions** | | |
| --- | --- | --- |
| - Available: 1 time per day, 6:00pm-10:00pm - Overview: Daily Diary Questions and Answers related to barriers and facilitators to physical activity - Completion: Participants can go back through survey after starting survey | | |
| **Question** | **Answers** | **Description for participant** |
| Good evening. We’d like to learn more about your day.   1. Overall, how stressful was your day? | - Extremely - Quite a bit - A little - Not at all | Thinking about the full day, the participant should rate how stressful it was. |
| 2. Which of these things have caused you stress today? Select all that apply. | - Job - Conflicts with Partner - Conflicts with Children - Other home conflicts - Friends - Finances - School - Other - No causes of stress today | Participants should mark all items that caused additional stress today. These may be small one-time conflicts or ongoing problems that they actively dealt with today. No causes of stress should be reported when they had no other causes. |
| 3. How much time did you engage in light activity today? Light activity includes light or minimal efforts (e.g., yoga, golf, easy walking) | - Hours__Minutes | Light activity involves physical activity that is minimal intensity with low effort. This may be an activity where the heart is pumping, but the participant is working at less than 50 percent of their maximum heart rate, making it safe for any individual of all ages and fitness levels to complete. Examples are provided, but the participant may consider other activities they perform. Consider the total amount though out the day, and report that amount. |
| 4. How much time did you engage in moderate activity today? Moderate activity gets your heart rate up to a high level but is not exhaustive (e.g., fast walking, tennis, easy swimming). | - Hours_minutes - *(Life data scroll option for each, 0-23 hours, 0-59 minutes)* | Moderate activity is exercise-like intensity activity. This may be an activity where the participant’s heart rate is up (or high), but you can generally speak to someone else or continue a conversation. General walking, like walking to the mailbox, may be considered a light activity. These activities may be daily activities, or structured. Examples are provided, but the participant may consider other activities they perform. Consider the total amount though out the day, and report that amount. |
| 5. How much time did you engage in vigorous activity today? Vigorous activity includes activities where your heart beats rapidly (e.g., running, vigorous bicycling, basketball). | - ___Hours___minutes - *(Life data scroll option for each, 0-23 hours, 0-59 minutes)* | Vigorous activity is the highest exercise intensity. This is where the participant’s heart rate is up (or high), and it is difficult to continue a conversation or speak in full sentences. These activities may be daily activities, or structured. Examples are provided, but the participant may consider other activities they perform. Consider the total amount though out the day, and report that amount. |
| 6. What are some things that got in the way of you being physically active today? (choose all that apply) | - Too tired - Feeling what I do doesn’t help - Impairment/Pain - No one to help me - Not interested - Embarrassment about my appearance - Lack of time - Bad weather - Other - N/A was active today | Participants should mark all items that interfered with physical activity engagement today. These may be small one-time conflicts or ongoing problems they actively dealt with during the day. No barriers to physical activity should be reported when they had no other causes. |
| 7. What was the major barrier to activity today? | - Too tired - Feeling what I do doesn’t help - Impairment/Pain - No one to help me - Not interested - Embarrassment about my appearance - Lack of time - Bad weather - Other - N/A was active today | Participants should identify the primary obstacle that interfered with participating in an activity today. Completion of activity should be reported. |
| 8. What are some things that made it easy to be physically active today? (choose all that apply) | - Free time - Being well rested - Pain relief - People to exercise with - Assistance - Mood and exercise enjoyment - Encouragement from others - Weather - Activities of daily living (i.e. housework, yardwork, etc.) - Manual labor job - Other - N/A no facilitators | Participant should mark all items that motivated them to be physically active during the day. No additional factors should be reported when they had no other causes. |
| 9. What was the biggest facilitator to activity today? | - Free time - Being well rested - Pain relief - People to exercise with - Assistance - Mood and exercise enjoyment - Encouragement from others - Weather - Activities of daily living (i.e. housework, yardwork, etc.) - Manual labor job - Other - N/A no facilitators | Participants should identify the primary factor that assisted with completion of activity today. |
| 10. How many servings of fruits and vegetables did you eat today? | - None 0 - 1-2 servings - 3-4 servings - 5+ servings | We are interested in the overall amounts of servings of fruits and vegetables consumed during the day. The participant should mark the total number of servings of fruits and vegetables |
| 11. Did you wear the tracker today? | - Yes - No - Don’t Know | The participant should report if they wore the activity tracker for the full day. **If there is difficulty wearing the tracker, please contact us.** |
| 12. Overall, how difficult was it for you to fill out the surveys TODAY? | - Not at all - A Little - Moderately - Quite a bit - Extremely | Considering the full day, the participant should consider how difficult it was to answer surveys. **If the participant is having significant problems with the prompting schedule or receiving prompts – please contact us.** |
| **Thank you for completing the survey!** | None |  |

| **Supplementary Table 4. Signal-Contingent Survey Questions** | | |
| --- | --- | --- |
| - Available: 2 times per day, sent randomly between 2 windows, 8:00am-12:00pm and 4:00-8:00pm. - Overview: Short Survey Question and Answers related to Daily Movement Behaviors Patterns and Context. - Completion: Participants can NOT go back through survey | | |
| Unlike the morning and evening questions, these questions are meant to be quick and focus on what the participant is feeling and doing at that moment. The participant should be prepared to answer all questions in a row, since the participant will not be able to go back. | | |
| **Question** | **Answers** | **Description for participant** |
| In these questions, we want to learn more about how YOU are doing right now.   1. Where are you located? | - Home – Indoors - Home – outdoors - Someone else’s home - Outdoors – not at home - Work - Car/Van/Truck/Vehicle - Childcare/School - Gym/ recreation center - Store/mall - Restaurant - Other | The participant should report where they are when the prompt went off. If the participant is in-between options, please choose the BEST option for their location. If they do not want to report the location, they may choose “other”. |
| 2. What are you doing right now? | - Eating - Reading - Work - Physical Activity - Screen-time 🡪 next question - Other | Participants should identify what kind of activity they are engaged in at that moment. |
| 3.  2a.) If screen-time, which devices are you using? | - TV - Computer - iPad/Tablet - Cellphone - Other | If the participant is using an electronic device, they should identify that particular device. |
| 4. How intense is your current activity? | - High (e.g., running, cycling fast, sports) - Medium (e.g., fast walking, skipping) - Low (e.g., easy walking, light effort) - Sitting, lying down - Standing | The participant should identify the intensity of their current state of physical activity. Examples have been provided, but the participant may be engaged in other activities. |
| 5. Are you performing this activity alone? | - Yes - No | The participant should report if they are actively participating in physical activity with another individual. |
| 6.  5a.) If no, who are you actively engaged with? (Select all) | - Friend - Spouse - Family member - Co-worker - Other | If the participant has indicated that they are currently performing an activity with another individual, the participant should mark all individuals that they are actively engaged in the activity with. |
| **Thank you for completing the survey!** | None |  |
